# Up regulated hippocampal insulin pathway and oxidative stress are related to opposite changes in olfaction and episodic memory in a compensatory metabolic syndrome model in rats

**DOI:** 10.3389/fncel.2026.1845387

**Published:** 2026-07-07

**Authors:** Pedro Isauro Clavel-Pérez, Bryan V. Phillips-Farfán, Luz Camacho-Castillo, Ana Laura Calderón-Garcidueñas, Karla Carvajal

**Affiliations:** 1 Laboratorio de Nutrición Experimental, Instituto Nacional de Pediatría, Mexico City, Mexico; 2 Departamento de Neuropatología, Instituto Nacional de Neurología y Neurocirugía, Manuel Velasco Suárez, Mexico City, Mexico

**Keywords:** insulin resistance, memory, metabolic syndrome, olfaction, oxidative stress

## Abstract

Insulin resistance, a major component of metabolic syndrome (MetS), is involved in phosphorylated tau and beta amyloid buildup, linking MetS to neurodegenerative processes, e.g., Alzheimer’s disease. One of the initial signs of neurodegeneration, in diseases such as Parkinson’s and Alzheimer’s, is olfactory dysfunction. Accordingly, in previous reports we found that high carbohydrate diet based on 30% sucrose in drinking water, increased visceral fat and insulin resistance, leading to an augment of oxidative stress and reduced brain energy metabolism, upregulating amyloidogenic genes (*APP* and *BACE1*) in hypothalamus and hippocampus. Thus, herein we comparatively evaluated the effect of sucrose induced MetS on episodic memory, olfaction, protein expression in amyloidogenic and insulin pathways, lipoperoxidation, antioxidant and BACE1 enzymatic activity between the hippocampus and olfactory bulb in male Wistar rats. Also, cellular structure of the olfactory bulb was analyzed. After 24 weeks of 30% sucrose consumption, we found preserved performance in a memory test; decreased hippocampal expression of APP and hyperactivation of the insulin pathway, low levels of lipoperoxidation, and high activity of antioxidant and BACE1 enzymes. Olfactory dysfunction was present in MetS rats, accompanied by morphological alterations in the olfactory bulb along with BACE1 increased activity. Thus, chronic high-sucrose consumption may prime the brain for neurodegenerative processes by modulating insulin signaling, oxidative stress homeostasis and amyloidogenic pathways in a region-dependent manner, with the olfactory system emerging as an early and sensitive target of metabolic dysfunction.

## Highlights

- Hippocampal insulin signaling and antioxidant defenses were enhanced in sucrose-fed rats during a compensated stage of metabolic syndrome.
- Activation of the hippocampal insulin signaling was associated with reduced APP expression and increased BACE1 activity in rats with metabolic syndrome.
- Sucrose-induced metabolic syndrome increased BACE1 activity without changes in protein expression, olfactory dysfunction and subtle cytoarchitectural alterations in the olfactory bulb, indicating early regional brain vulnerability.

## 1 Introduction

Metabolic Syndrome (MetS) is a group of conditions including insulin resistance (IR), dyslipidemias, visceral adiposity, obesity, and high blood pressure that raise the risk of coronary heart disease, type 2 diabetes mellitus (T2D) and stroke (Noubiap et al., 2022). Rat models of MetS induced by a high sucrose diet (HSD) display features similar to human MetS, like ROS production, IR, hypertriglyceridemia, abdominal obesity and hypertension (Rodríguez-Correa et al., 2020).

Many studies have shown that MetS and neurodegenerative diseases are robustly linked (Akhtar and Sah, 2020). Obesity (Ma et al., 2020), IR (Femminella et al., 2021) or T2D (Madhusudhanan et al., 2020) augment the chance of suffering neurodegenerative diseases, each on their own, and the risk increases when they occur together (Pugazhenthi et al., 2017). In particular, MetS and/or its constituents make it more likely to develop Alzheimer’s disease (AD), the most important cause of dementia, linking the metabolic alterations present in MetS with some underlying cellular mechanisms of neurodegeneration (Arnold et al., 2018). In this sense, it has been demonstrated that, IR may increase the activity of β-site APP cleaving enzyme 1 (BACE1), leading to a buildup of amyloid-β (Aβ) oligomers, which together, in turn, could impair insulin signaling, thus creating a vicious circle (Ohno, 2025). Aβ is a component of neuritic plaques, which characterize AD.

In a previous work, we found that MetS induced by a HSD reduces energy metabolism in the brain by decreasing AMPK expression in the hippocampus and creatine kinase (CK) activity in the hippocampus and hypothalamus (Camacho-Castillo et al., 2021). MetS also promotes oxidative stress (OS) in these brain areas and enhances BACE1 and amyloid precursor protein (APP) gene expression, likely increasing amyloidogenesis (Camacho-Castillo et al., 2021). Thus, to further investigate the impact of such metabolic alterations in central nervous system functions related to cognitive decline, we treated animals for 24 weeks with a HSD to explore the functional effect on memory and olfaction. We compared the cellular mechanisms affected in two brain regions that rule such functions, hippocampus and olfactory bulb, taking into account that smell dysfunction is one of the early signs of prodromal neurodegenerative diseases such as AD and Parkinson’s disease and may precede or prone memory loss (Kreisl et al., 2018; Bathini et al., 2019).

## 2 Methods

### 2.1 Subjects

Twenty-four Wistar male rats, 2 months old, weighing 250 to 300 g, were the subjects of experimentation. They were acquired from the animal facility of the Biomedical Research Institute of the National Autonomous University of Mexico. Female rats were not included in the experiment because they naturally are resistant to develop diet-induced MS alterations, via estradiol antioxidant protection of OS derived from hypercaloric diets (Busserolles et al., 2002), or via a delay in exhibiting diet-induced MS alterations (Maric et al., 2022). The sample size was calculated according to previous publications (Balderas-Villalobos et al., 2013), however, not all of the animals were used for all experiments. Uneven numbers of rats were used for the control and experimental animals given that, in some cases, samples were exhausted or not available due to the nature of some tests, some others were excluded according to the established criteria. Instead, the respective n was indicated at every specific procedure in figures and graphs. Upon arrival, 3–4 rats were transferred to polycarbonate boxes (dimensions 43 cm × 53 cm × 20 cm). Rats were housed in the institutional animal facility with 12-h light–dark cycles, 19–22 °C temperature and 40–60% relative humidity. Animals had a 5-day habituation phase before starting treatment. Rat management was carried out in accordance with Official Mexican law (NOM-062-ZOO-1999), institutional regulations and the NIH Guide for the Care and Use of Laboratory Animals (National Research Council (US) Committee for the Update of the Guide for the Care and Use of Laboratory Animals, 2011). The experimental protocol was approved by the Institutional Research Commission and the Committee for the Care and Use of Laboratory Animals (registry number INP 025/2015 and 056/2022). All procedures were conducted in accordance to ARRIVE guidelines and the principles of the 3R’s to minimize animal use (Percie du Sert et al., 2020).

### 2.2 Diet

**Figure 1:**
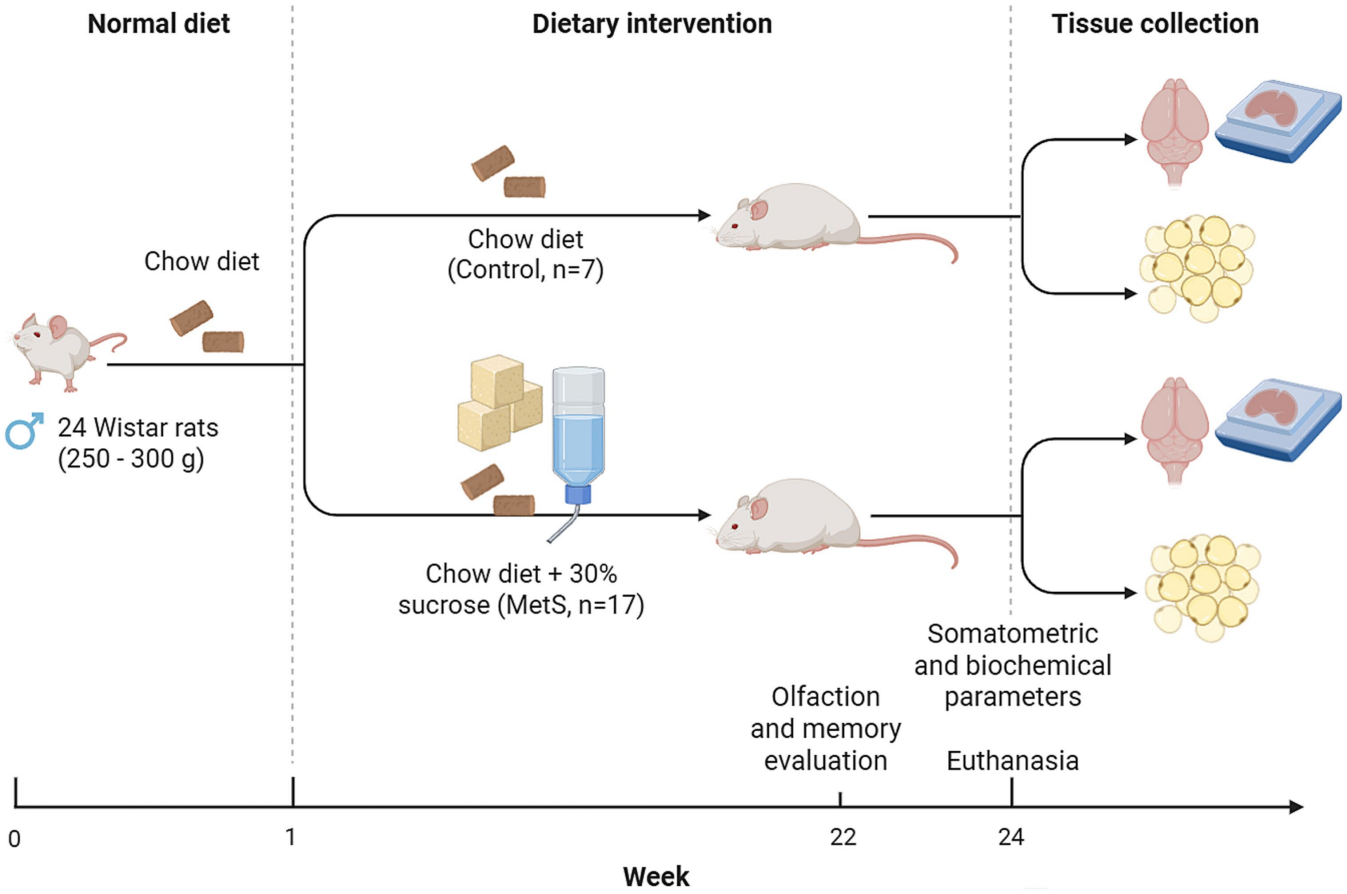
Timeline showing the experimental design. All of the behavioral evaluations were made starting at 12 p.m. at light phase. Mouse High Fat Diet Experimental Timeline from Clavel (2026). Created in BioRender. https://BioRender.com/9qt0048. License: Student plan-academic. Source: BioRender.com.

After acclimatization, rats were assigned to the control or MetS group. No randomization was done because rats were highly homogenous. The first group received standard rodent diet (Autoclavable Rodent Diet 5,010; LabDiet, United States) and free access to water (*n* = 7). The MetS group (*n* = 17) received the same standard diet and 30% sucrose diluted in their drinking water to induce MetS (Figure 1) for 24 weeks. Water drunk and food consumption was weekly monitored. The number of animals per group is consistent with previous studies using hypercaloric diets to induce MetS, where control groups range from 6 to 10 animals and experimental groups are even larger than 20 animals, to account for variability and multiple endpoints (Camacho-Castillo et al., 2021; Rodríguez-Correa and Carvajal, 2024).

### 2.3 Euthanasia and sample collection

At the end of week 24, after behavior tests, rats were deep anesthetized with 200 mg/kg sodium pentobarbital administered in the peritoneum to ensure complete loss of consciousness. Blood was collected by cardiac puncture followed by exsanguination to achieve. The hippocampus and olfactory bulb were subsequently dissected from a subset of animals, rapidly frozen in N_2_, and stored at −70 °C for later biochemical determinations. For histological analysis, a different lot of rats were perfused with ice-cold saline solution (200 mL approximately), then the whole brain was extracted and post-fixed in 4% paraformaldehyde solution. In animals assigned for histological analysis, some samples were not obtained and thus some biochemical or tissue assay were not available.

### 2.4 MetS determination

There is currently no consensus to determine MetS in rodents. Based on the criteria for diagnosing MetS in humans (Alberti et al., 2009), several somatometric and biochemical parameters were measured in control and MetS groups. To determine whether the experimental group presented metabolic syndrome after 24 weeks, the following parameters were evaluated in both groups: body weight, fasting glucose, serum triglycerides and adipose tissue weight to validate the condition of MetS or control status. For the group receiving sucrose, if serum triglycerides were below 200 mg/dL, the animals were excluded as MetS animals. Likewise, for the control group, if triglycerides were above 200 mg/dL, then rats were excluded. As well, animals showing significant loss of weight and clear signs of pain or unwellness, were discarded from the study. For convenience, we used some representative samples from the MetS group to confirm and validate the already and widely reported changes in serum insulin levels and homeostatic model index for evaluating IR (HOMA-IR) (Camacho-Castillo et al., 2021; Balderas-Villalobos et al., 2013; Rodríguez-Correa and Carvajal, 2024; Gómez-Viquez et al., 2023).

#### 2.4.1 Body weight

Throughout the 24 weeks of treatment, the weekly body weight of rats was recorded using a digital scale.

#### 2.4.2 Fasting blood glucose

After a 5-h fast (from 7:00 a.m. to 12:00 p.m.) and before anesthetic administration, blood glucose levels were measured by tail vein puncture using a portable glucometer and disposable strips (FreeStyle Optium; Abbott, United States).

#### 2.4.3 Blood triglycerides

In the awake and fasted animals, a blood drop obtained from tail vein puncture was used to measure triglyceride levels using a portable equipment and disposable strips (Accutrend GCT; Roche, United States).

#### 2.4.4 Serum insulin levels

The blood was centrifuged at 3,500 rpm at 4 °C for 15 min to obtain serum. An ELISA kit for determining insulin in rats (ALPCO, United States) was used.

#### 2.4.5 HOMA-IR

The homeostatic model index for evaluating IR (HOMA-IR) was calculated from plasma glucose and insulin values. HOMA-IR is indicative of the feedback loop between glucose and insulin levels. Although it was developed for humans (Matthews et al., 1985), its use has been validated in rats (Antunes et al., 2016).

#### 2.4.6 Adipose tissue

After euthanasia, adipose tissue from retroperitoneal fat pads was collected and weighed using an analytical balance. The weight of adipose tissue was normalized to the size of the tibia (g/cm).

### 2.5 Behavioral tests

At weeks 22 and 23 of treatment, behavioral testing was performed in control and MetS groups. Odor habituation/dishabituation and the hidden chocolate tests were performed to assess olfactory function. The novel object recognition (NOR) test was performed to assess declarative memory.

#### 2.5.1 Habituation/dishabituation tests

The purpose of this test is to assess whether a rodent is capable of perceiving an odor and if it can also distinguish when there is an odor change. This procedure was modified from a reported protocol (Yang and Crawley, 2009). One day prior to the task, rats from both groups were moved to the room where the tasks would be performed. Each rat was habituated in a new, clean box with an odorless swab. The next day, rats were presented with a successive sequence of different odors impregnated in a cotton swab. Each odor was presented 3 times for 2 min, with an inter-trial interval of 1 min. Rats were first presented with a swab moistened with water (odorless). Then, a swab with vanilla extract (Molina^®^, Mexico) diluted to a concentration of 1:100. Then, a social odor obtained by rubbing the cotton swab against the bedding of a cage housing unfamiliar rats. Habituation was defined as a progressive decrease in olfactory investigation toward the repeated presentation of the same olfactory stimulus. On the other hand, dishabituation was considered as the restoration of olfactory investigation toward a novel odor. The time (in seconds) during which rats showed olfactory behavior toward the swab (movement of whiskers and nose toward the swab) was recorded.

#### 2.5.2 Buried food test

The purpose of this simple paradigm is to determine if a rat can perceive an olfactory stimulus. A previously reported protocol was used (Yang and Crawley, 2009). Rats were given a test food (sugar free Turin^®^ chocolate, 5-gram tablet) 2 days before evaluation. It was verified for rats to have gnawed or eaten the test food the next morning. After an overnight fast, rats were placed in a clean box for 5 min. After this habituation time, rats were placed in another box for 1 min and the chocolate was buried within the sawdust of the habituated box. Rats were placed back, on the opposite end from where the chocolate was placed, and the time it took for the rat to discover the piece of food was recorded. The test was stopped if a rat did not find the chocolate in the next 5 min.

#### 2.5.3 Novel object recognition test (NOR)

This task is based on the tendency of rodents to explore novel objects. The protocol used was based on a previous study (Antunes and Biala, 2012). The test consists of three phases. In the first phase, rats were familiarized with two totally identical objects placed inside a clean box. Each rat was positioned in front of the pair of objects for 10 min and the exploration time was recorded. This test was repeated 4 times, with an interval between tests of 10 min. The second phase consisted in the storing and consolidation of the information, being away from the objects for 24 h. The next day at phase three, a completely different object replaced one of the identical objects. Objects were cleaned with 70% ethanol at the end of each test with each rat. The exploration time toward the objects was recorded during all phases of the experiment.

### 2.6 Protein extraction

The frozen tissues (olfactory bulb and hippocampus) were mechanically homogenized using a polytron. The tissues were suspended in cold lysis solution containing (in mM): HEPES 50, KCl 50, EDTA 1, EGTA 1, β-glycerol-phosphate 5, Triton X100 0.1%, NaF 50, NaPPi 5, DTT 1, PMFS 0.2, sodium orthovanadate 1, plus a cocktail of proteases inhibitors (complete, Roche). Homogenates were centrifuged at 12,000 rpm, 4 °C for 30 min. After this, the supernatant was recovered and stored at −70 °C for later use.

### 2.7 Protein quantification

Protein concentration was quantified using Lowry’s method (Lowry et al., 1951) using albumin as standard.

### 2.8 Antioxidant enzymes and BACE1 activities determination

Homogenates from the olfactory bulb and hippocampus were used for enzymatic determinations. Glutathione reductase and superoxide dismutase (SOD) activities were measured using kits ADI-900-159 and ADI-900-157, respectively (Enzo Life Sciences, Inc.). For absorbance or fluorescence readings, a spectrophoto-fluorometer was used (Modulus II Microplate, Turner BioSystems).

#### 2.8.1 Catalase activity

Samples were diluted 1:50 with phosphate–buffered saline PBS. Fifty microliters of diluted samples were added to a 96-well plate, followed by 50 μL of 40 μM H_2_O_2_, and incubated for 10 min at room temperature. Then, 100 μL of cocktail buffer containing ABTS (1.09 mM), HRP (26 μM) and PBS buffer were added. After 10-min of incubation, absorbance was measured at 420 nm. A catalase standard curve (0–4 U/mL) was generated. Samples and standard curves were analyzed in duplicates, and enzyme activity was normalized to total protein concentration.

#### 2.8.2 Glutathione reductase activity

A standard curve ranging from 0.0005 to 0.02 U/50 μL was prepared. Fifty microliters of undiluted samples or standards (in duplicate) were added to a 96-well plate along with 100 μL of master mix containing oxidized glutathione and glutathione reductase buffer. The reaction was initiated by adding 50 μL of NADPH to the plate. Absorbance at 340 nm was measured every minute for 11 min to monitor NADPH oxidation. Enzymatic activity was normalized to total protein concentration.

#### 2.8.3 Superoxide dismutase activity

Samples were diluted 1:50 in PBS and assayed in duplicate. A standard curve ranging from 0.1 to 10 U/μL was prepared in duplicate. Twenty-five microliters of diluted samples or standard were added to a 96-well plate containing 1X SOD buffer. Subsequently, 150 μL of master mix (WST-1 reagent and xanthine oxidase) were added to each well. The reaction was initiated by adding 25 μL of xanthine solution. Absorbance at 450 nm was measured every minute for 10 min. Enzyme activity was normalized to total protein concentration.

#### 2.8.4 BACE1 enzymatic activity

BACE1 activity was determined using β-Secretase activity assay kit (565,785, Calbiochem, Merck). Protein samples were adjusted to a concentration of 300 ng/μL with buffer lysis. 50 μL of diluted sample were added to a 96-well plate, followed by 46 μL of reaction buffer and 4 μL of BACE1 substrate. The plate was incubated at 37 °C, and fluorescence was measured every 10 min for 100 min (excitation wavelength of 335–355 nm, emission wavelength of 495–510 nm).

For all of the enzymatic assays, rates were obtained by calculating the slope from a linear regression of the changes of absorbance or fluorescence over time; samples were interpolated into a standard curve when available.

### 2.9 Malondialdehyde determination (MDA)

Samples (200 μg of protein) were placed in glass tubes. They were treated with an antioxidant solution (methanol + 4% butylated hydroxytoluene), diluted in phosphate buffer (KH_2_PO_4_ 150 mM, pH 7.4) and incubated at 37 °C for 30 min with gentle shaking. Acetic (20%) and thiobarbituric (1.6%) acids were added to the samples, which were then incubated at 100 °C for 1 h. The reaction was stopped by incubating the samples on ice for 10 min. After adding KCl (2%) and butanol (2 mL), the samples were centrifuged at 1500 rpm for 2 min. The organic phase was obtained and loaded (200 μL) onto wells (96-well plate). The fluorescence was determined at 515/553 nm excitation/emission (Modulus II Microplate, Turner BioSystems). Tetraethoxypropane (0.01 mM) received the same treatment and was used as a calibrating standard.

### 2.10 Western blotting

After quantification, 60 μg of total protein from homogenates were loaded into SDS-PAGE gels at 10%. Electrophoresis was performed at 90 v, for 30 min, then 120 v, for 140 min. Proteins were transferred to PVDF membranes and probed with the following antibodies: Anti-amyloid precursor protein + beta amyloid antibody (ab 126,873, Abcam); anti-BACE1 (ab2077, Abcam); anti-insulin receptor (phospho Y972, ab5678, Abcam); anti-insulin receptor (β-Subunit) clone CT-3 (#05–1,104, Millipore); phospho-Akt 1 (ser 473, #9018, Cell signaling); anti-AKT (610,860, BD) all diluted 1:1000 and anti-actin (A2103, Sigma) 1:5000 was used as a loading control. The protein was detected by chemiluminescence, using a Chemidoc-XRS instrument and analyzed with Quantity-One software (Bio-Rad Laboratories Inc.).

### 2.11 Cresyl violet staining

Paraformaldehyde fixed brains were dehydrated through graded ethanol solutions and paraffin-embedded for microtome sectioning. Staining protocol was adapted from a previous described method (Zhang and Xiong, 2014). Coronal sections of the olfactory bulb (20 um thickness) were obtained from the medial region (interaural 3.4 mm). Sections were mounted on gelatin-coated slides, deparaffinized and immersed in 0.1% cresyl violet solution for 15 min. Afterwards, slides were rinsed in distilled water followed by ethanol to remove excess staining. Stained sections images were obtained using the MoticEasyScan One (Motic). Morphological analyses were performed using ImageScope software (Leica Biosystems). For pyramidal cell density, a 500 μm region was delimited in the mitral cell layer of the olfactory bulb (posterior part of olfactory bulb). The same region was analyzed for all the slides. Images were analyzed at 10X objective.

### 2.12 Statistical analysis

The buried food and NOR trials, somatometric and biochemical parameters, BACE1 and antioxidant enzyme activity, as well as olfactory bulb morphology were analyzed using Mann–Whitney *U* tests. To analyze the habituation/dishabituation test, intra-group Mann–Whitney-Wilcoxon tests were used. The results are expressed as mean ± standard error of the mean (SEM). A *p* ≤ 0.05 was considered statistically significant. However, when the *p* value was close to significance, its numerical value was shown. Data processing, statistical analysis and graphs were made in GraphPad Prism software 8.0. Statistical power was estimated for some representative biochemical and behavior endpoints to evaluate the sensitivity and robustness of the chosen analyses. Most comparisons achieved power values exceeding 80%, suggesting that the analysis had a reasonable capacity to detect biologically meaningful differences between groups (Supplementary Table 1).

## 3 Results

### 3.1 Somatometric and biochemical parameters

Administration of 30% sucrose in the drinking water for 24 weeks resulted in a significant increase in body weight, visceral adiposity, and circulating triglyceride levels. Fasting glucose levels remained unchanged; however, insulin levels were significantly elevated, leading to an increased HOMA-IR index (Table 1) as widely reported for this model (Camacho-Castillo et al., 2021; Balderas-Villalobos et al., 2013; Rodríguez-Correa and Carvajal, 2024; Gómez-Viquez et al., 2023). Collectively, these findings indicate that the high-carbohydrate diet induced a MetS phenotype characterized by systemic IR in a compensatory stage, in which normoglycemia is maintained through hyperinsulinemia.

**Table 1:** Somatometric and biochemical parameters at week 24 of HSD.

Parameter	Control	MetS
Final weight (g)	568.6 ± 11.84 *n* = 7	678.2 ± 20.39^**^ n = 17
Glucose (mg/dL)	84.86 ± 2.41 *n* = 7	90.82 ± 4.25 *n* = 17
Insulin (mU/dL)	2.73 ± 0.18 *n* = 4	4.56 ± 0.87^*^ n = 6
HOMA-IR	0.56 ± 0.06 *n* = 4	1.06 ± 0.2 *n* = 6
Triglycerides (mg/dL)	162 ± 15.65 *n* = 7	279.8 ± 22.05^***^ n = 17
VAT (g)	14.85 ± 1.3 *n* = 7	29.41 ± 3.04^**^ n = 17
VAT/Tibia (g/cm)	3.52 ± 0.5 *n* = 7	6.96 ± 0.72^**^ n = 17
Caloric intake (Kcal/rat/day)	74.32 ± 1.28 *n* = 7	106.5 ± 3.05 *N* = 17

VAT, visceral adipose tissue. *p ≤ 0.05, **p ≤ 0.01, ***p ≤ 0.0001, Control vs. MetS. Mean ± SEM.

### 3.2 Habituation/dishabituation test

**Figure 2:**
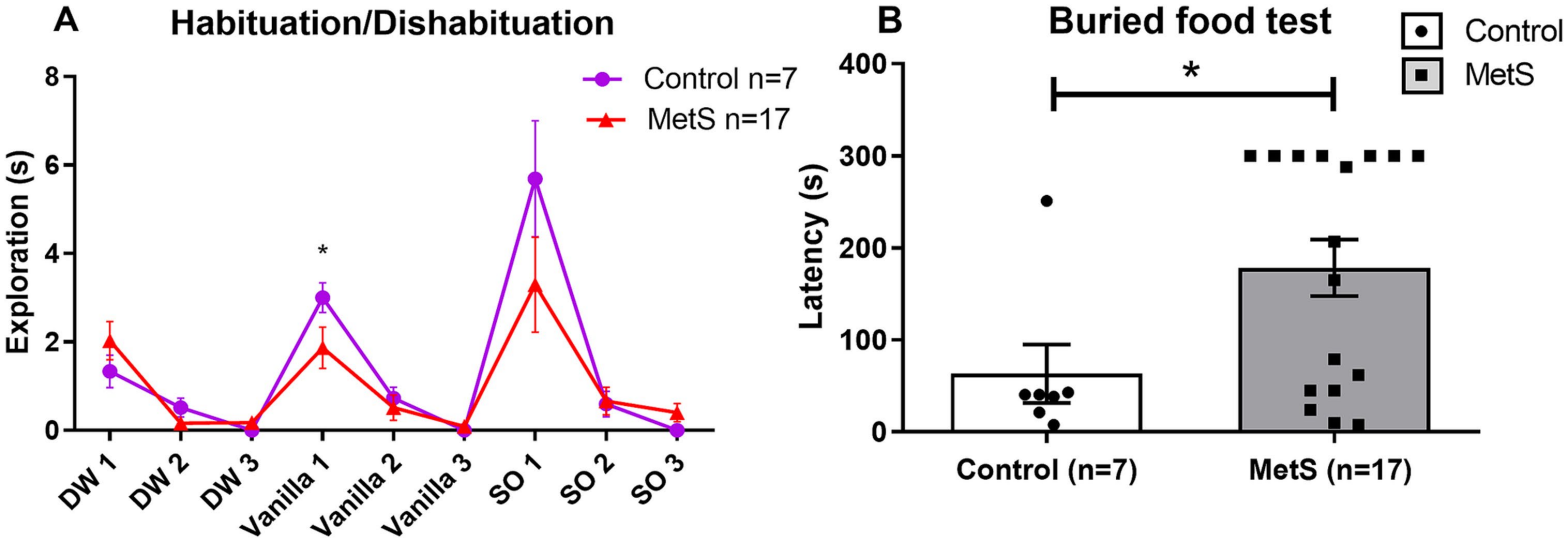
Performance in olfactory tests. **(A)** Olfaction times for the different olfactory stimuli. Each stimulus was presented for 2 min for three trials, with an inter-trial interval of 1 min. *p ≤ 0.05 Control vs. MetS. Mean ± SEM. DW, distilled water; SO, social odor. **(B)** Average latency to unearth the hidden pellet. *p ≤ 0.05.

Both groups habituated to the repeated exposure to olfactory *stimuli*, as reflected by the gradual reduction in exploration times. Similarly, both groups increased exploration when presented with a different olfactory stimulus, which demonstrates dishabituation (Figure 2A). Several processes are involved in performing this task: detection of a novel olfactory stimulus, decrease in olfaction upon stimulus repetition (habituation) and the recognition of another novel olfactory stimulus (dishabituation). MetS did not alter task performance, although rats fed with an HSD had lower exploration time in the first vanilla stimulus (*p* < 0.05; Figure 2A).

### 3.3 Buried food test

After overnight fasting, in which both groups only had access to tapwater, MetS rats had a higher latency to find the hidden chocolate piece (Figure 2B). This test is dependent on smell, so a greater latency to find the hidden food could indicate failure or delay in capturing the odor stimulus and then locating its source. Thus, there could be signs of an olfactory alteration in MetS animals, which prevented the correct performance of this task.

### 3.4 NOR test

**Figure 3:**
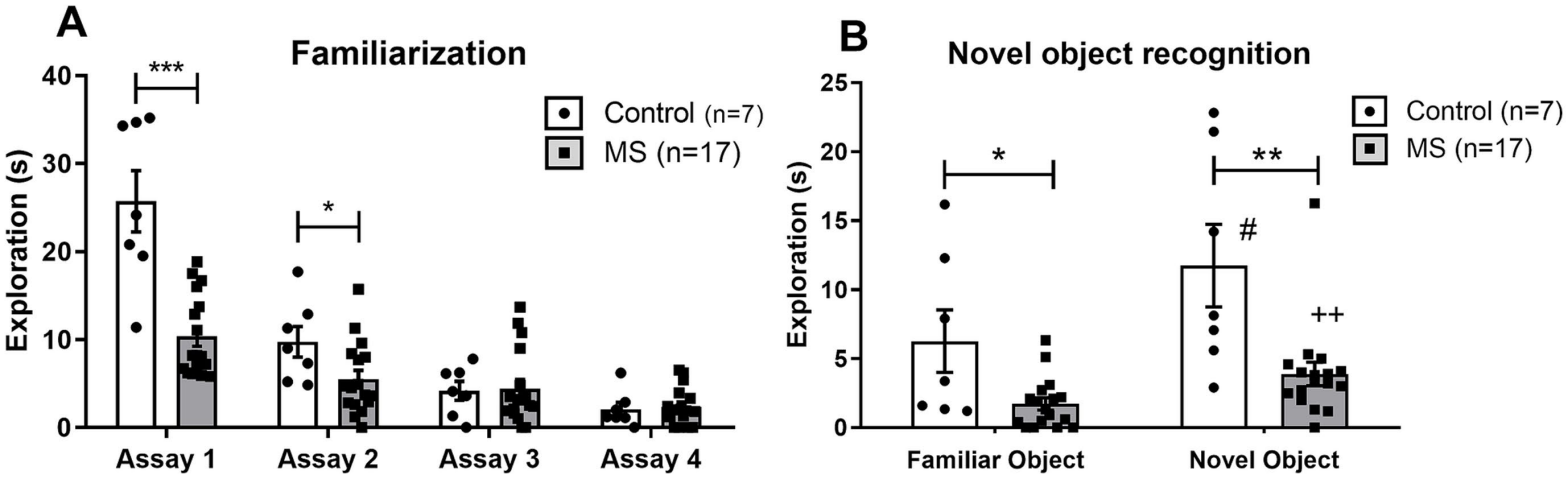
Novel object recognition (NOR) test performance. **(A)** Exploration times toward the familiar objects during the familiarization phase. **(B)** Exploration times toward the familiar and novel objects during the test trial. *p ≤ 0.05, **p ≤ 0.01 Control vs. MetS; ^#^*p* ≤ 0.05 novel vs. familiar Control, ^++^*p* ≤ 0.01 novel vs. familiar MetS. Mean ± SEM.

The NOR test was performed to determine the effect of MetS on memory. During the first phase (familiarization), exploration of the familiar objects decreased with successive trials (Figure 3A). In the test trial (third phase), 24 h after presentation of two identical objects (second phase), rats were placed in front of the familiar object and a totally novel object. Both groups had a greater preference to explore the novel object than the familiar object (Figure 3B). Even though MetS rats had shorter explorations times across objects in the test trial than controls, they still had a preference to explore the novel object (Figure 3B). Discrimination index, a method to evaluate the exploration relative to total time of rats exploring the objects, showed no statistically differences between groups (Control 52.5 ± 8.5, MetS 60.7 ± 6.5, Mean ± SEM).

### 3.5 Hippocampal protein expression

**Figure 4:**
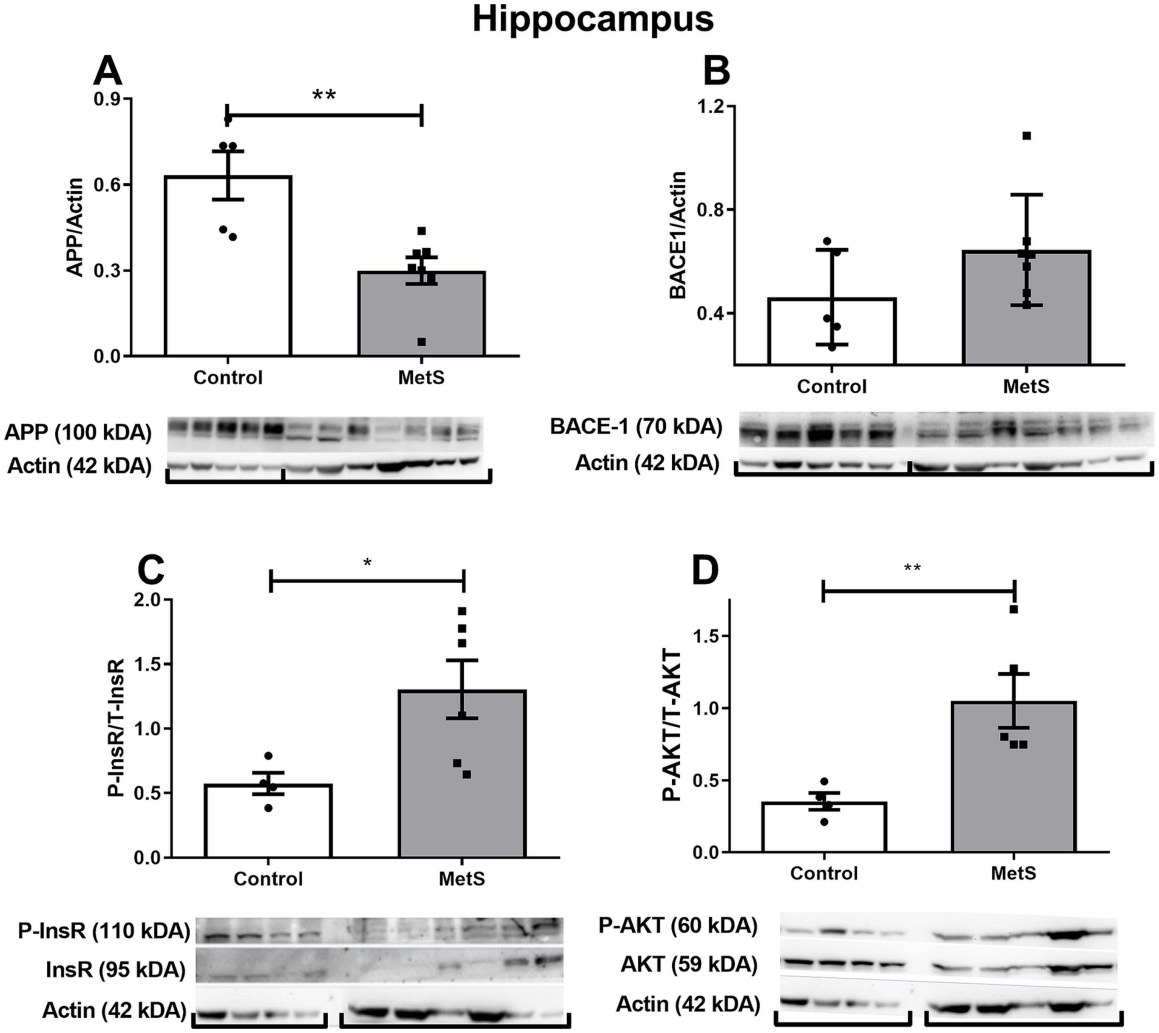
Protein expression of: **(A)** APP (Control *n* = 5, MetS *n* = 7), **(B)** BACE1 (Control *n* = 5, MetS *n* = 7), **(C)** P-InsR, T-InsR (Control *n* = 4, MetS *n* = 6) **(D)** P-AKT and T-AKT (Control *n* = 4, MetS *n* = 5) in the hippocampus. *p ≤ 0.05, **p ≤ 0.01, Control vs. MetS. Mean ± SEM. APP, amyloid precursor protein, BACE1, β-site APP cleaving enzyme 1; P-InsR, phosphorylated insulin receptor; T-InsR, total insulin receptor; P-AKT, Phosphorylated AKT; T-AKT, total AKT.

The APP and BACE1 protein expression was evaluated. The MetS group exhibited a significant reduction in APP expression (Figure 4A), whereas BACE1 protein levels were not significantly altered (Figure 4B). Given the state of peripheral hyperinsulinemia on MetS rats, brain insulin signaling was studied. InsR and AKT were selected representing key upstream and downstream nodes of the canonical insulin-signaling pathway. Their combined evaluation allows detection of insulin resistance-associated alterations, including compensatory activation or downstream signaling deficiency (Al Haj Ahmad et al., 2022). Phosphorylation levels of the InsR and protein kinase B (AKT), were elevated in MetS animals (Figures 4C,D), indicating enhanced compensatory activation of the hippocampal insulin pathway. No differences were observed in total InsR or AKT protein expression.

### 3.6 Olfactory bulb protein expression

**Figure 5:**
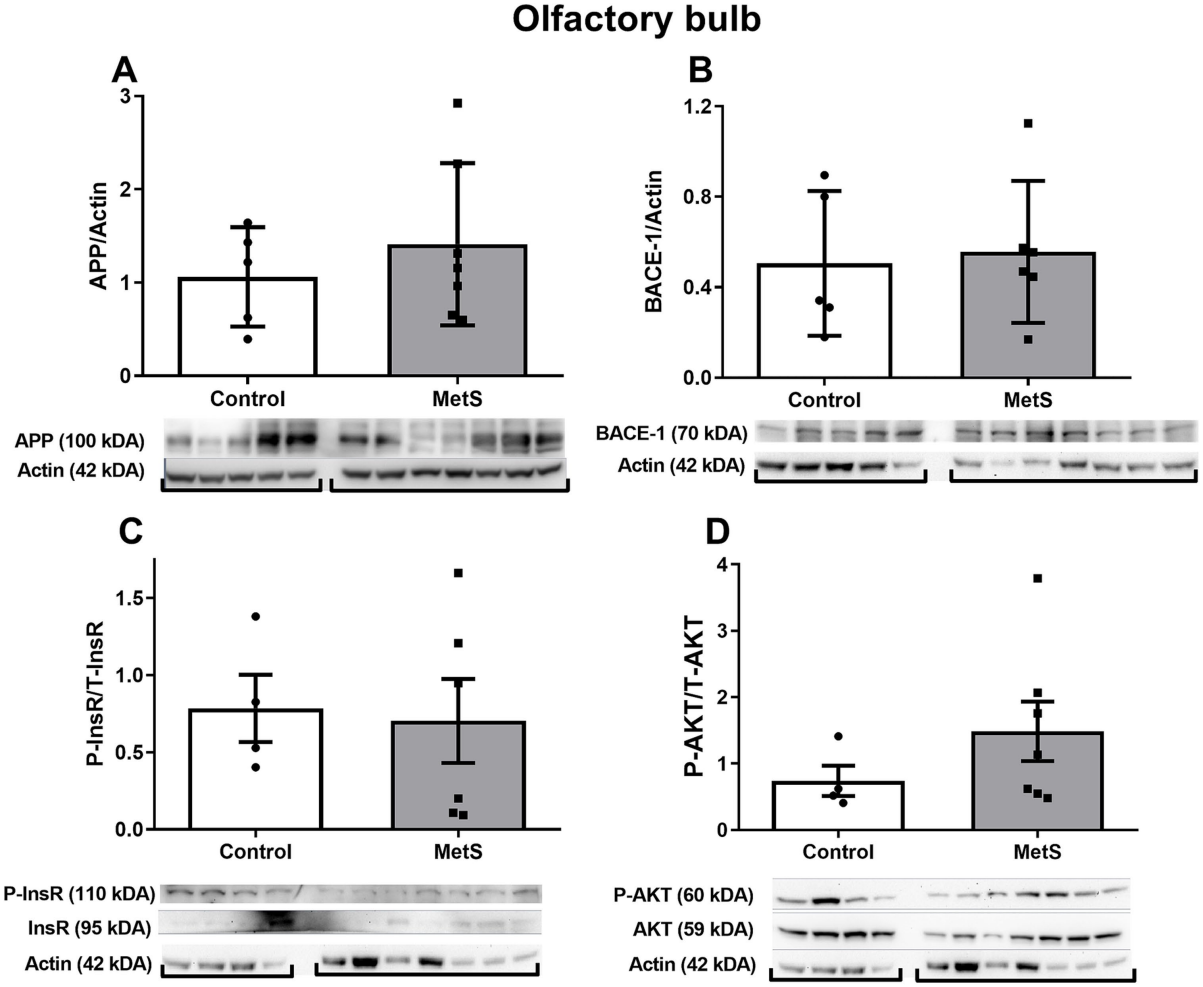
Protein expression of **(A)** APP (Control *n* = 5, MetS *n* = 7), **(B)** BACE1 (Control *n* = 5, MetS *n* = 6), **(C)** P-InsR, T-InsR (Control *n* = 4, MetS *n* = 6), **(D)** P-AKT and T-AKT in the olfactory bulb. Mean ± SEM. APP, amyloid precursor protein; BACE1, β-site Amyloid Precursor Protein-Cleaving Enzyme 1; P-InsR, phosphorylated insulin receptor; T-InsR, total insulin receptor; P-AKT, phosphorylated AKT; T-AKT, total AKT.

Clinical research indicates that olfactory dysfunction appears even decades before memory loss (Kreisl et al., 2018; Bathini et al., 2019), positioning the olfactory system as one of the earliest affected regions during neurodegenerative processes. Accordingly, the olfactory bulb represents a sensitive site to detect early molecular alterations associated with neurodegeneration (Rey et al., 2018). In this context, we evaluated APP and BACE1 protein expression in the olfactory bulb (Figures 5A,B), to investigate whether early dysregulation of APP metabolism was occurring in this brain region. No significant differences were observed between MetS and the control group (Figures 5C,D). Similarly, insulin signaling appeared unaltered, as InsR and AKT expression and their phosphorylation levels remained the same as controls. Notably, this regional response contrasts with the hippocampus from the same animals. No changes were found on total InsR and AKT protein levels.

### 3.7 BACE1 activity in the hippocampus and olfactory bulb

**Figure 6:**
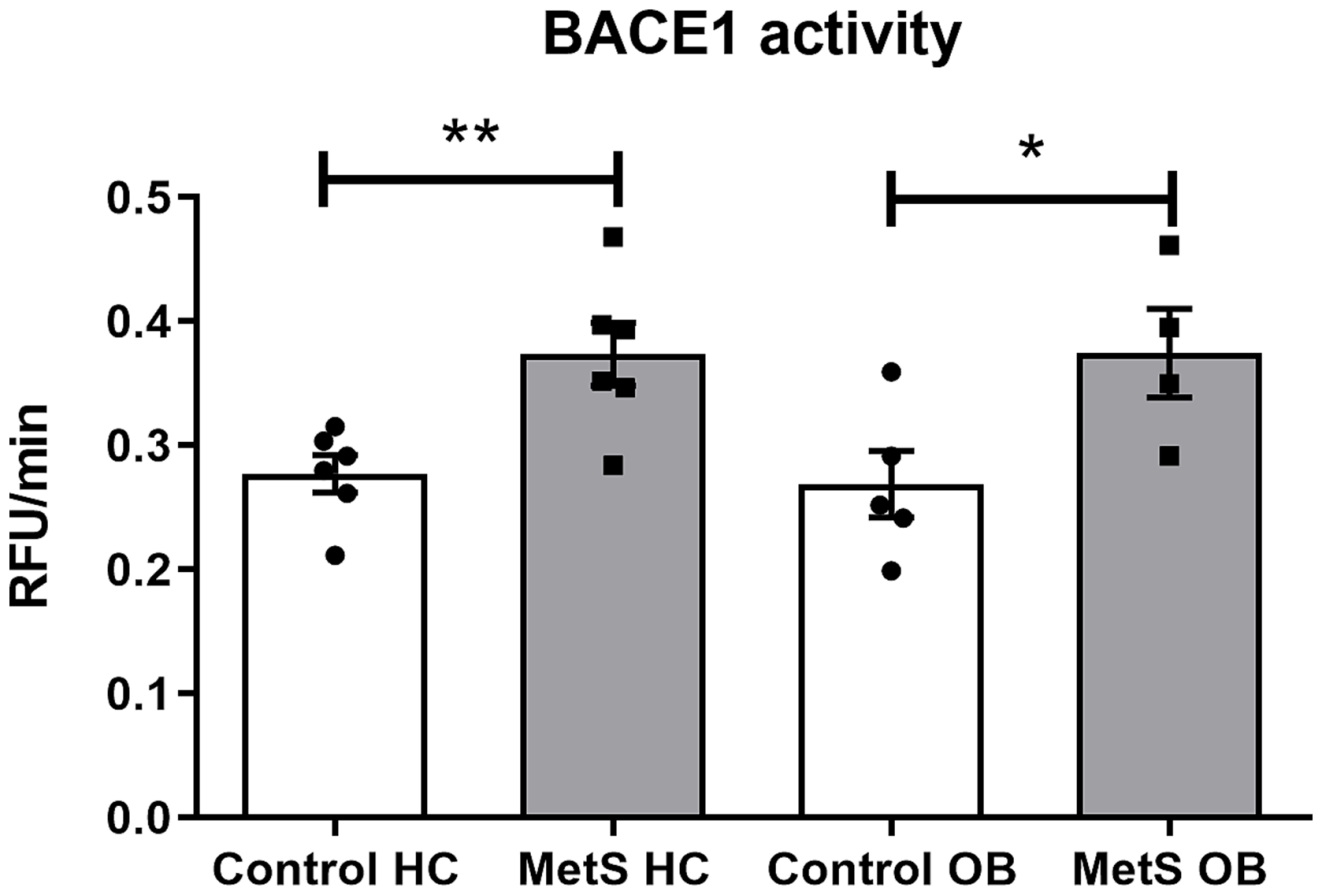
BACE1 enzymatic activity in the hippocampus (Control, MetS, *n* = 6) and olfactory bulb (Control *n* = 5, MetS *n* = 4) of control and MetS rats. Mean ± SEM. *p ≤ 0.05, **p ≤ 0.01, Control vs. MetS, Mann–Whitney *U* test. HC, hippocampus; OB, olfactory bulb.

To determine whether MetS modulates the functional activity of the amyloidogenic pathway beyond protein expression, BACE1 enzymatic activity was evaluated in hippocampus and olfactory bulb lysates. Rats with MetS exhibited a significant increase in BACE1 enzymatic activity in both hippocampus and olfactory bulb compared to control animals (Figure 6). Notably, this increase occurred despite the unchanged BACE1 protein expression in MetS rats (Figures 4B, 5B).

### 3.8 Oxidative stress markers in the hippocampus and olfactory bulb

**Figure 7:**
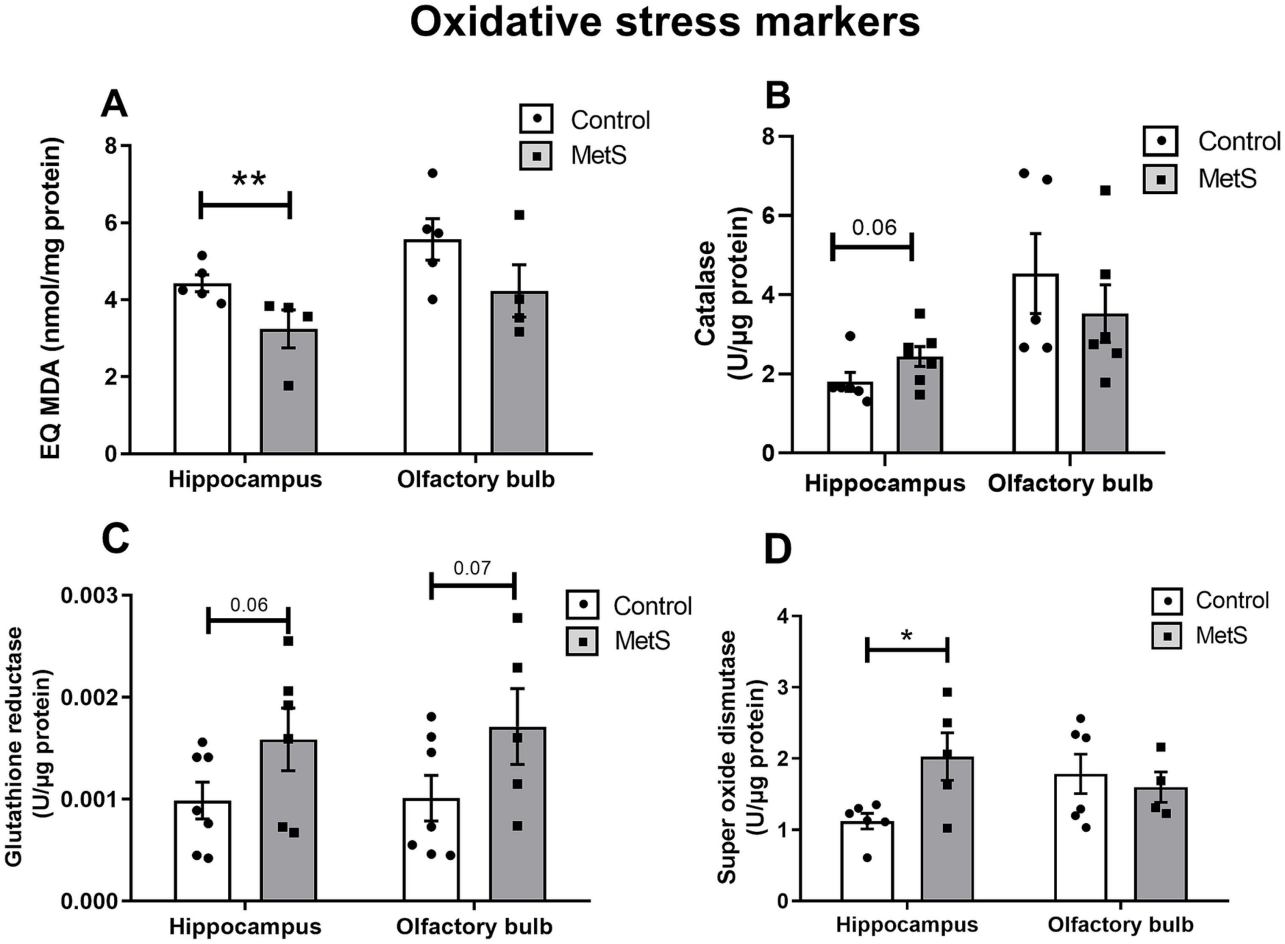
Oxidative markers in hippocampus and olfactory bulb. **(A)** MDA determination (lipoperoxidation) in the hippocampus and olfactory bulb (Control *n* = 5, MetS *n* = 4). Antioxidant enzyme activities: **(B)** Catalase (HC Control *n* = 6, MetS *n* = 7; OB Control *n* = 5, MetS *n* = 6); **(C)** Glutathione reductase (HC Control *n* = 7, MetS *n* = 6; OB Control *n* = 7, MetS *n* = 5) and **(D)** Super oxide dismutase (HC Control *n* = 6, MetS *n* = 5; OB Control *n* = 6, MetS *n* = 4) in the hippocampus and olfactory bulb. *p ≤ 0.05. **p ≤ 0.01. Mean ± SEM. MDA, malondialdehyde.

The presence of OS was determined by measuring MDA, an end product generated by lipid peroxidation and by measuring antioxidant enzymatic activities. Interestingly, the hippocampus of MetS rats had lower MDA levels, but no differences were found in the olfactory bulb (Figure 7A). Catalase (Figure 7B), glutathione reductase (Figure 7C) and superoxide dismutase (SOD) (Figure 7D) activities were determined in the hippocampus and olfactory bulb. The MetS group showed an increased SOD activity in the hippocampus (Figure 7D). The other enzyme activities tended to augment in MetS animals, but did not reach statistical significance (Figures 7B,C).

### 3.9 Neuronal cytoarchitecture in olfactory bulb

**Figure 8:**
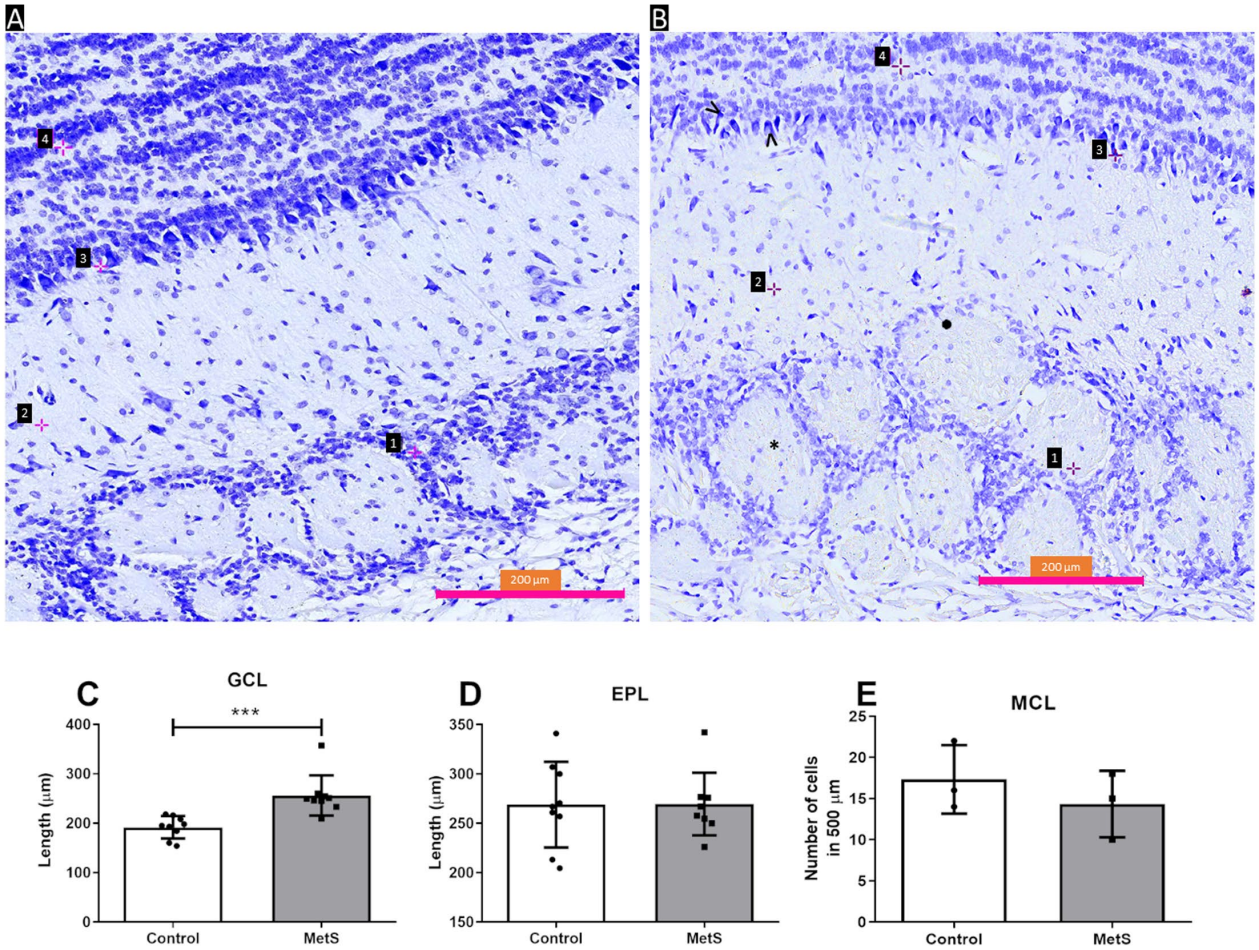
Cytoarchitectural alterations in the olfactory bulb of rats with MetS. Representative cresyl violet-stained sections of the olfactory bulb from Control (*n* = 3) **(A)** and MetS (*n* = 3) **(B)** rats. The main cell layers are indicated: glomerular layer (GCL, 1), external plexiform layer (EPL, 2), mitral cell layer (MCL, 3), and granular layer (Ma et al., 2020). In MetS rats, glomeruli show fused boundaries (*) and swollen periglomerular cells (●). The GCL is significantly wider in MetS rats **(C)**, with no apparent differences in EPL thickness **(D)** or mitral cell count **(E)**. Neurons with shrunken somata and pyknotic or veiled nuclei (^) are also observed in the MCL. ***p ≤ 0.001. Mean ± SEM.

Given that MetS rats had an altered olfactory exploration in the buried food test, we conducted histological analysis in olfactory bulb sections with cresyl violet staining. MetS rats exhibited structural alterations potentially involving both cytoarchitectural remodeling and neurodegenerative processes compared to control tissue (Figure 8A). Specifically, there was a partial loss of laminar definition in the glomerular layer, as the boundaries between adjacent glomeruli appeared indistinct (Figure 8B). Notably, the glomerular layer was wider and irregular in MetS than the control group (*p* < 0.001, Figure 8C), a change that correlated with swollen periglomerular neurons compared to control slides. External plexiform cell layer (EPL, Figure 8D) and mitral cell layer showed no differences in pyramidal neurons density, but exhibited morphologic abnormalities (thinner, darker neurons with a veiled nuclei) in MetS rats (Figures 8B,E).

## 4 Discussion

In a previous report derived from our lab, 30% sucrose consumption for 22 weeks leads to several metabolic alterations, including increased adipose tissue, elevated triglycerides levels, hyperinsulinemia and a higher HOMA-IR index, while fasting blood glucose levels remained unchanged (Camacho-Castillo et al., 2021). In this work, extending sucrose consumption for an additional 2 weeks period resulted in a mild further increase in final body weight (Table 1). However, this weight gain was less pronounced than that caused by high-fat diets (HFD) models or genetic MetS models (Rivière et al., 2016). This difference in body weight gain suggests a more severe metabolic disturbance in HFD-fed rats. Nevertheless, rats fed a HSD still exhibit significant alterations in lipid metabolism, including increased adipose tissue mass. Such changes may promote adipocyte hypertrophy, leading to local inflammation and dysregulated adipokine production, including leptin and adiponectin (Pugazhenthi et al., 2017).

### 4.1 HSD impairs olfaction, sparing recognition memory

Sucrose consumption resulted in diminished olfactory memory and reduced olfactory perception. Similar findings have been reported in mice fed with a high-fructose diet, which exhibited longer latencies in the buried food test (Rivière et al., 2016), and in spontaneous T2D models showing increased retrieval latency and reduced exploration in habituation/dishabituation tasks (Lietzau et al., 2018). Notably, fructose-induced metabolic syndrome develops more rapidly, producing a more severe metabolic phenotype, characterized by marked hypertriglyceridemia, hyperinsulinemia, and glucose intolerance (Rodríguez-Correa et al., 2020). In contrast, our HSD-induced model represents an earlier and less severe stage of metabolic dysfunction because normoglycemia is maintained, consistent with a compensatory phase of systemic IR (Rodríguez-Correa et al., 2020). Importantly, the observed olfactory impairment appears to be subclinical, emerging during this compensatory stage rather than in overt T2D. These findings suggest that subtle sensory deficits may arise early in MetS, potentially reflecting neural adaptations associated with compensatory insulin activation.

In contrast, recognition memory was not affected in MetS animals. Nonetheless, other studies have shown that 35% sucrose consumption for 9 weeks impairs object and spatial recognition (Lemos et al., 2016). However, in that previous work the authors did not report metabolic parameters beyond body weight, preventing direct comparison with our model. Importantly, as long-term memory undergoes systems consolidation and becomes distributed across cortical networks, it exhibits greater resistance to neuronal damage compared to short-term memory, which relies on transient neural activity and is therefore more susceptible to disruption (Squire et al., 2015). Short-term memory represents a sensitive domain for detecting early cognitive alterations associated with metabolic syndrome and warrants further investigation in future studies.

### 4.2 MetS decreases APP expression and hyperactivates insulin pathway in the hippocampus

After 24 weeks of a HSD, hippocampal APP expression was reduced. In this study, we observed up-regulation of the insulin pathway in the hippocampus, possibly driven by systemic hyperinsulinemia. No significant differences in APP and BACE1 protein expression or insulin pathway activation were found in the olfactory bulb of MetS rats, suggesting regional IR in the brain, since InsR and AKT in the olfactory bulb remained non-responsive to peripheral hyperinsulinemia. Previous work from our group reported MetS-induced increases in APP and BACE1 gene expression, in the hypothalamus and hippocampus in rats with 22 weeks of 30% sucrose feeding (Camacho-Castillo et al., 2021). In the present work, although we did not measure gene expression, we presume that genomic changes other than protein expression may be occurring in our model, since two extra weeks of 30% sucrose feeding exacerbates somatometric parameters, and it could worsen amyloidogenic gene expression. Further research needs to be conducted to confirm this.

Previous studies have shown that activation of the insulin pathway plays an important role in the regulation of amyloid pathway. Insulin signaling has been reported to downregulate the transcription of pro-amyloidogenic genes, including APP and BACE1 (Pandini et al., 2013). This regulatory mechanism has been associated with inactivation of glycogen synthase kinase 3β (GSK3β), a key component of the insulin signaling cascade that has been implicated in driving amyloidogenic genes transcription (Pandini et al., 2013). Indeed, insulin deficiency induced by streptozotocin has been shown to increase BACE1 protein, resulting in elevated Aβ40 and Aβ42 in the brains of transgenic 5XFAD mice (Devi et al., 2012).

In the present study, despite the increased phosphorylation of InsR and AKT observed in the hippocampus, our results suggest that insulin resistance may occur at downstream levels of the signaling pathway, potentially involving GSK3β at least in this time frame. Therefore, further studies are required in our MetS model to evaluate the phosphorylation status and activity of this kinase. Given that BACE1 activity represents a key regulatory step in APP amyloidogenic processing, and is known to be modulated by oxidative stress, we next evaluated whether metabolic syndrome alters BACE1 enzymatic activity in brain regions relevant to early neurodegeneration.

### 4.3 Increased BACE1 activity in rats with early MetS

BACE1 is the principal enzyme of the amyloidogenic pathway; its activation under OS conditions promotes APP cleavage and consequently reduces total APP levels (Tong et al., 2005; Guglielmotto et al., 2009). For this reason, we assessed BACE1 enzymatic activity in rats with MetS. Exposure to an HSD significantly increased BACE1 activity in both the hippocampus and olfactory bulb. This elevation is consistent with the reduced hippocampal APP protein levels observed, which could be evidence of enhanced amyloidogenic processing.

Enzymatic activity was measured under maximal conditions to obtain an integrated readout of BACE1 regulation. This approach captures the cumulative impact of post-translational mechanisms (including phosphorylation and oxidative modifications) on enzyme function (Araki, 2016). Notably, BACE1 activity was increased despite comparable protein expression levels, indicating that functional changes are not driven solely by protein abundance. Rather, this dissociation likely reflects the combined influence of multiple regulatory mechanisms, including post-translational oxidative modifications that may be driven by the exposition to sustained OS imposed by MetS. Future studies are necessary to dissect the individual contributions of these kinds of modification such as phosphorylation, underlying BACE1 regulation in this context.

In a previous work, we reported that 22 weeks of a HSD induced both systemic and central OS, along with increased BACE1 gene expression in the hippocampus (Camacho-Castillo et al., 2021). Importantly, in the present work we found that the olfactory bulb exhibited elevated BACE1 activity without prior evidence of insulin pathway compensation. This finding reinforces the concept that the olfactory system is metabolically vulnerable, where early functional and structural alterations precede classical hippocampal pathology.

### 4.4 MDA levels and antioxidant enzyme activities show a compensatory response to MetS in brain regions

Given that BACE1 activity is modulated by OS, and that HSD induces oxidative imbalance (Camacho-Castillo et al., 2021), we evaluated antioxidant enzymes activity and lipid peroxidation in the hippocampus and the olfactory bulb. Notably, our results suggest the activation of a compensatory antioxidant response, more pronounced in the hippocampus and, to a lesser extent, in the olfactory bulb. These findings indicate an early adaptive response that may counterbalance oxidative damage.

Previous studies have reported increased lipoperoxidation and reduced enzymatic antioxidant enzyme activity in the hippocampus following a high carbohydrate-high fat diet (Alzoubi et al., 2013), although metabolic parameters were not described. Additionally, Żebrowska et al. (2020) demonstrated region-specific responses to HSD-induced OS, with decreased glutathione peroxidase and SOD activities but increased catalase activity in the hypothalamus, while the cerebral cortex remained unaffected. These observations underscore the heterogeneity of redox regulation across brain regions.

Collectively, our data indicate that during early MetS, despite established peripheral OS (Gómez-Viquez et al., 2023), the brain engages an adaptive antioxidant response, potentially delaying overt oxidative damage at the central level. Importantly, our findings extend previous evidence by showing that OS not only influences BACE1 gene expression (Camacho-Castillo et al., 2021) but also modulates its enzymatic activity.

### 4.5 The hippocampus responds to a systemic compensatory MetS state

Our findings indicate that after 24 weeks of 30% sucrose diet, rats’ brains exhibit a compensated stage of MetS characterized by hippocampal insulin-pathway hyperactivation accompanied by modulation of OS markers. Supporting this idea, previous work from our group using the same MetS model demonstrated that sucrose-fed rats maintained greater treadmill running capacity after 8 weeks of training (Rodríguez-Correa and Carvajal, 2024) indicating preserved functional plasticity in response to systemic hyperinsulinemia. These observations align with reports showing that early hyperinsulinemic states are accompanied by increased basal AKT activity in peripheral tissues, reflecting a compensatory attempt to maintain glucose homeostasis (Liu et al., 2009).

Evidence from IR models indicates that a sustained brain insulin-pathway hyperactivation can become maladaptive. Reports indicate that HFD or genetic induced obesity in mice and primates with T2D produces an increased basal activation of AKT/aPKC in the hippocampus and increased Aβ peptides levels and tau phosphorylation, indicating a shift toward neurodegeneration (Sajan et al., 2016). In contrast, enhancing hippocampal insulin signaling through exercise improves cognition in HFD fed mice, supporting a neuroprotective role of insulin signaling (Park et al., 2019). Our findings suggest that in an early and less severe MetS phase, hippocampal insulin pathway hyperactivation is tied with antioxidant defenses, regulating lipoperoxidation, and BACE1 enzymatic activation.

### 4.6 MetS disrupts cytoarchitecture in the olfactory bulb

Cresyl violet staining revealed mild but consistent structural alterations in the olfactory bulb of rats under HSD. Previous evidence has shown a correlation between the consumption of dietary fats (commonly used to induce obesity) and structural alterations in the olfactory epithelium (Chelette et al., 2022). Specifically, a HFD, whether provided ad libitum or calorie-matched, leads to a loss of olfactory sensory neurons in the olfactory epithelium, thereby reducing the number of neuronal projections that reach the olfactory bulb. Although the aforementioned research did not evaluate morphological changes within the olfactory bulb, it is reasonable to presume that the glomerular layer may be affected, given that this region constitutes the first relay station for axonal projections arriving from the olfactory epithelium. However, HFD produces more chronic metabolic disturbances (Rodríguez-Correa et al., 2020), reflecting a more advanced stage of MetS. In our study, we observed changes in the morphology of the olfactory bulb that may correlate with functional alterations (olfactory dysfunction) at an early and still compensated stage of MetS induced by a HSD.

These structural findings are relevant and compatible with early alterations in the olfactory bulb, a structure characterized by high cellular turnover and plasticity, indicating putative subclinical signs of valuable use for opportune identification of central nervous system dysfunction, particularly when metabolic diseases are present. Nevertheless, future studies approaching cell death markers are necessary to clarify further the contribution of MetS to neurodegeneration.

Olfactory dysfunction is a strong predictor for dementia onset, because it is present years before any other cognitive impairment, such as memory loss (Kreisl et al., 2018; Bathini et al., 2019). Even though Aβ peptides deposition in the olfactory pathway are considered the underlying process to early olfactory dysfunction, as seen in AD patients and transgenic AD rodent models (Ayala-Grosso et al., 2015; Kovács et al., 2001; Wesson et al., 2010), underlying pathology prior to Aβ deposition (such as increased active microglia and cytokine release) is indicative that inflammatory processes occur before neuritic plaque deposition (Welikovitch et al., 2020). OS and IR generate neuronal function impairment (Potukuchi et al., 2018; Tyagi et al., 2020) and can accelerate amyloidogenic processes in the brain (Verdile et al., 2015). Human and murine APP sequences differ in three amino acids, hindering murine Aβ deposition and neuritic plaque formation; nonetheless, they possess the complete amyloidogenic pathway and the respective surrounding regulatory mechanisms, allowing a more precise visualization of the impact of metabolic disturbances upon neurodegenerative processes (Xu et al., 2015). Herein, we demonstrate that even in the natural absence of amyloid deposition in rat brain, early metabolic imbalance (OS and IR) directly affects olfactory function in MetS, which could act synergistically with Aβ plaques when they are present, as it occurs in other mammals species, such as humans, and thus accelerate neurodegeneration, implicating the interaction between obesity and neurodegenerative processes, evident at early stages of the disease.

Other cellular degenerative processes may be elicited just for OS and IR. For example, apoptosis was present in olfactory sensory neurons expressing human APP (Cheng et al., 2011). Considering that our results indicate structural alterations in the glomerular layer of the olfactory bulb in MetS rats, it remains to be determined whether apoptotic mechanisms contribute to these morphological changes. We propose that IR is present in the olfactory bulb of rats with MetS, since despite systemic hyperinsulinemia, insulin activation remained unchanged even at a fasting state. In contrast, hyper-activation of the insulin pathway was observed in the hippocampus.

## 5 Conclusion and further considerations

In conclusion, a HSD induces an early and compensated stage of MetS characterized by systemic hyperinsulinemia, dyslipidemia, and preserved normoglycemia. Systemic IR elicited compensatory adaptations in the hippocampus, including insulin-pathway hyperactivation, and a compensatory response of cellular antioxidant system. Despite this apparently protective state, BACE1 enzymatic activity is increased and APP protein levels are reduced, indicating that the initial phase of amyloidogenic processing may be enhanced during early stages of MetS. In contrast, the olfactory bulb did not exhibit these molecular changes, yet it displayed early functional and structural alterations consistent with observed olfactory deficit. These findings suggest that the olfactory pathway may represent a metabolically sensitive neural circuit in which functional and structural alterations emerge earlier than in other brain regions. Further studies are required to characterize the specific cellular and molecular mechanisms underlying this damage, including potential contributions of inflammatory signaling, electrophysiological alterations within the olfactory circuit, and other metabolic stress-related pathways. Moreover, a deeper characterization of amyloidogenic regulation needs to be evaluated in this model, including accumulation of Aβ peptides, while also evaluating tau phosphorylation status to relate MetS status to the formation of neurofibrillary tangles.

## Data Availability

The raw data supporting the conclusions of this article will be made available by the authors, without undue reservation.
