# Supplementary material for: Up regulated hippocampal insulin pathway and oxidative stress are related to opposite changes in olfaction and episodic memory in a compensatory metabolic syndrome model in rats
Source: Front Cell Neurosci. 2026 Jul 7;20:1845387. doi: 10.3389/fncel.2026.1845387 (PMC13384890; doi:10.3389/fncel.2026.1845387)
Supplement: Supplementary file 1 [file Table_1.PDF]

Supplementary table 1. Statistical power analysis for the Mann-Whitney U test.

Parameter	Sample size (Control / MetS)	Mean (Control / MetS)	Standard deviation (Control / MetS)	Pooled SD	Reported p-value	Effect size (Cohen's d)	Statistical Power (ARE, 1-tailed, α=0.05
**Final weight**	7 / 17	568.6 / 678.2	31.32 / 84.06	73.53	0.011	1.49	93.8%
**Insulin**	4 / 6	2.73 / 4.56	0.37 / 2.14	1.72	0.01	01.06	45.2%
**Triglycerides**	7 / 17	162.0 / 279.8	41.40 / 90.90	80.20	0.0005	1.47	93.2%
**Adipose tissue**	7 / 17	14.85 / 29.41	3.44 / 12.57	10.96	0.0038	1.33	87.2%
**Buried food test**	7 / 17	63.19 / 178.40	83.83 / 126.70	117.15	0.013	0.98	60.3%
**NOR F-A1**	7 / 7	25.72 / 2.07	9.262 / 2.023	6.61	0.94	3.58	99.9%
**NOR F-N Ctrl**	7 / 7	4.41 / 9.45	4.169 / 6.311	5.35	0.78	0.94	52.2%
**NOR F-N MetS**	17 / 17	1.72 / 3.89	1.827 / 3.487	2.78	0.94	0.78	70.2%
**AKT activation**	4 / 5	0.35 / 1.05	0.11 / 0.41	0.32	0.0159	2.16	83.60%
**Superoxide dismutase activity**	6 / 5	1.12 / 2.02	0.26 / 0.74	0.54	0.0411	1.67	67.00%

NOR F-A1: Novel object recognition test, familiarization assay 1; NOR F-N: Familiar vs Novel.

To contextualize the magnitude of the experimental effects and evaluate the sensitivity of the sample sizes chosen for the experiments, we calculated the post-hoc effect size and statistical power using the Asymptotic Relative Efficiency (ARE) method in the G*Power software for the Mann-Whitney U tests. Because non-parametric tests do not assume a specific underlying distribution, power was estimated by scaling the nominal sample sizes to an effective sample size relative to an equivalent parametric Student's t-test using an efficiency factor of 0.955, following the framework established by Faul et al. (2007). Asymmetrical sample sizes were accounted for by calculating the pooled standard deviation to derive Cohen's d effect sizes. All power metrics were determined based on a directional (one-tailed) hypothesis with a significance threshold of $\alpha = 0.05$. The majority of the evaluated parameters displayed a robust statistical power exceeding the standard 80% threshold. For parameters where power fell below 80%, large effect sizes were still observed; this localized reduction in power is attributable to the high biological variance and smaller sample sizes inherent to those specific cohorts. Ultimately, the high statistical power achieved across the primary metrics confirms the overall mathematical robustness and validity of the presented dataset.

Reference

Faul F, Erdfelder E, Lang AG, Buchner A. G*Power 3: a flexible statistical power analysis program for the social, behavioral, and biomedical sciences. Behav Res Methods. 2007 May;39(2):175-91. doi: 10.3758/bf03193146.
